# A side-by-side evaluation of Llama 2 by meta with ChatGPT and its application in ophthalmology

**DOI:** 10.1038/s41433-024-02972-y

**Published:** 2024-02-12

**Authors:** Mouayad Masalkhi, Joshua Ong, Ethan Waisberg, Nasif Zaman, Prithul Sarker, Andrew G. Lee, Alireza Tavakkoli

**Affiliations:** 1 University College Dublin School of Medicine, Belfield, Dublin, Ireland; 2 Department of Ophthalmology and Visual Sciences, University of Michigan Kellogg Eye Center, Ann Arbor, MI USA; 3 Human-Machine Perception Laboratory, Department of Computer Science and Engineering, University of Nevada, Reno, Reno, NV USA; 4 Department of Ophthalmology, University of Cambridge, Cambridge, UK; 5 Center for Space Medicine, Baylor College of Medicine, Houston, TX USA; 6 Department of Ophthalmology, Blanton Eye Institute, Houston Methodist Hospital, Houston, TX USA; 7 The Houston Methodist Research Institute, Houston Methodist Hospital, Houston, TX USA; 8 Departments of Ophthalmology, Neurology, and Neurosurgery, Weill Cornell Medicine, New York, New York, USA; 9 Department of Ophthalmology, University of Texas Medical Branch, Galveston, TX USA; 10 University of Texas MD Anderson Cancer Center, Houston, TX USA; 11 Texas A&M College of Medicine, Texas, USA; 12 Department of Ophthalmology, The University of Iowa Hospitals and Clinics, Iowa City, IA USA

**Keywords:** Epidemiology, Eye manifestations

## Introduction

Llama 2, a product of Meta, represents the latest advancement in open-source large language models (LLMs). It has been trained on a massive dataset of 2 trillion tokens, which is a significant increase compared to its predecessor, Llama 1 [1]. Its ability to understand and generate language, combined with its optimized transformer architecture and fine-tuning methods, make it a potentially valuable resource in various fields.

Llama 2 is available in a variety of sizes, with parameters ranging from 7 billion to 70 billion, and includes both pretrained and fine-tuned versions [1]. The fine-tuned models, known as Llama 2-Chat, have been optimized for dialogue applications [1]. The Llama 2 model uses an optimized transformer architecture, which is a network architecture based solely on attention mechanisms [1].

The architecture of Llama 2 is based on an optimized transformer model, a network architecture that relies solely on attention mechanisms [1]. This allows the model to focus on different parts of the input sequence when generating an output, thereby enhancing its language understanding and generation capabilities [1].

In the field of ophthalmology, LLMs are approaching expert-level knowledge and reasoning skills and have the potential to provide valuable medical advice and assistance in areas where access to expert ophthalmologists is limited [1]. Another notable application of Llama 2 is Code Llama, a code generation model built on Llama 2, which has been trained on 500 billion tokens of code [1].

**Fig. 1:**
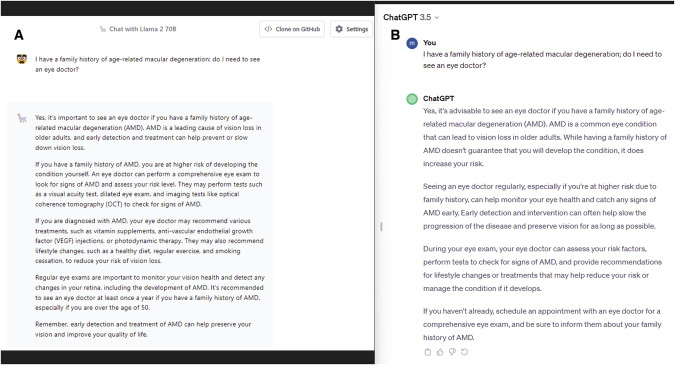
A Side-by-Side Evaluation of Llama 2 by Meta with ChatGPT and Its Application in Ophthalmology. Last revision on November 21, 2023. **A** Output generated by Llama 2 from the prompt “I have a family history of age-related macular degeneration; do I need to see an eye doctor?” **B** Output generated by ChatGPT. The responses generated by both Llama 2 and ChatGPT mentioned the importance of visiting an eye specialist. Furthermore, both models suggested appropriate advice and protective steps that can be taken lower the chances of AMD progression.

**Fig. 2:**
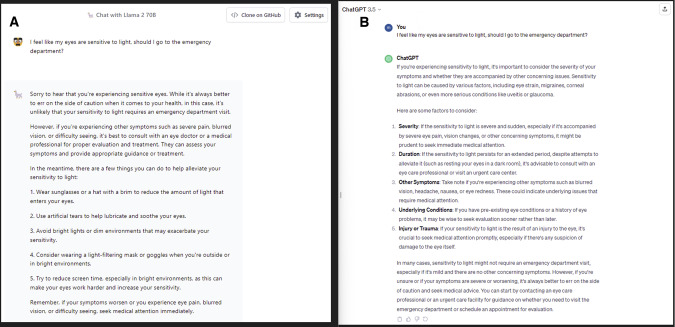
A Side-by-Side Evaluation of Llama 2 by Meta with ChatGPT and Its Application in Ophthalmology. Last revision on November 21, 2023. **A** Output generated by Llama 2 from the prompt “I feel like my eyes are sensitive to light, should I go to the emergency department?”. **B** Output generated by ChatGPT.

We prompted both the AI chatbots that a patient was reporting a sensitivity to light in both eyes, and if they should attend the emergency department (Fig. 1). Both Llama 2 and ChatGPT correctly recommended to attend the emergency department and the importance of seeking urgent medical care. We found that the response generated by Llama 2 focused more on ways of alleviating the pain, which ChatGPT focused more on the different causes of photophobia. These AI-generated outputs were both specific, and appropriate (Fig. 2).

**Fig. 3:**
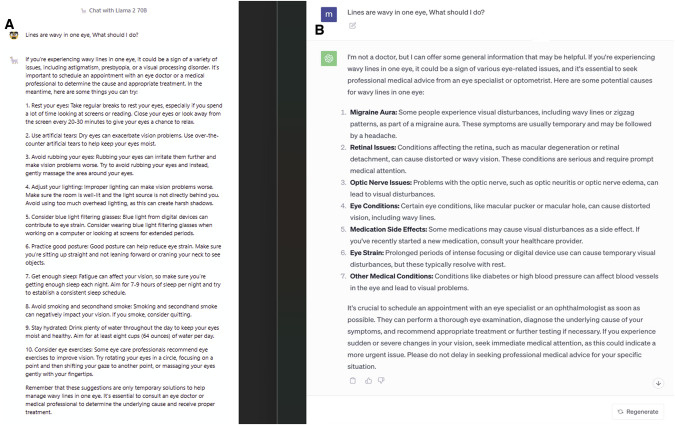
A Side-by-Side evaluation of Llama 2 by Meta with ChatGPT and its application in ophthalmology. Last revision on November 21, 2023. **A** Output generated by Llama 2 from the prompt “Lines are wavy in one eye, What should I do?”. **B** Generated by ChatGPT.

In addition, we prompted both AI chatbots what to do if a patient is seeing that lines appear wavy in one eye (Fig. 3). Generating a suitable suggestion from this input is challenging as the reason for this blurry vision can be a result from refractive errors, age-related conditions, eye injuries, eye infections, or more serious conditions.

**Fig. 4:**
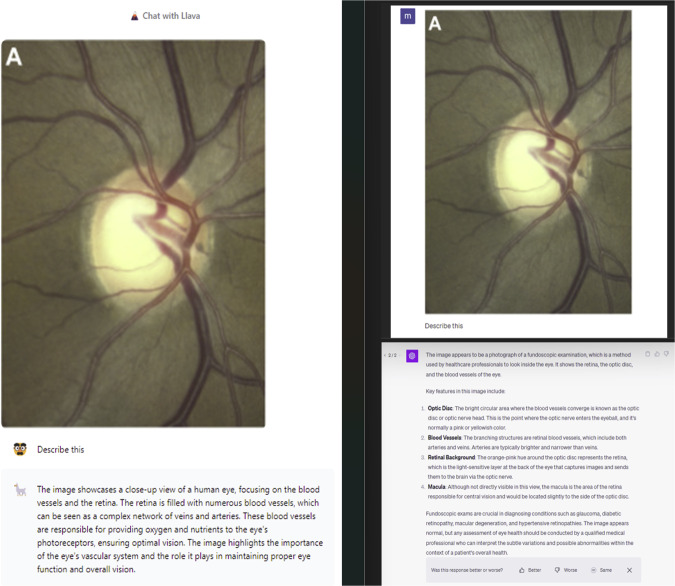
A Side-by-Side evaluation of Llama 2 by Meta with ChatGPT and its application in ophthalmology. Last revision on November 21, 2023. Reprinted without changes from Waisberg E, Georgiou M. Unilateral congenital non-syndromic retinal vessel dilation and tortuosity. Am J Ophthalmol Case Rep. 2021 Sep;23:101160. 10.1016/j.ajoc.2021.101160 under Creative Commons Attribution - Non Commercial (unported, v3.0) License.

Llama 2 provided a comprehensive list of steps and actions to performs to address the possible reason for seeing blurry lines. ChatGPT mentioned the importance of seeking professional medical help as soon as possible, especially if sudden or severe vision changes are experienced. ChatGPT’s response focused more on the different causes for those symptoms such as issues in the retina, optic nerve, a migraine aura, among others,

Finally, we examined Llama 2’s image analysis capabilities by providing a fundus image of glaucomatous cupping in the right optic nerve (Figure 5). [2]. Llama 2 stated that it is not a medical expert prior to providing any information. Llama 2 correctly identified the retina and blood vessels, however, did not specify the optic disc or make any remark on the optic disc cupping. GPT-4 on the other hand correctly identified the optic disc, retina, and the blood vessels. However, GPT-4 incorrectly mentioned that this image appears to be normal (Fig. 4).

### Limitations

The Llama 2 model, a powerful LLM renowned for its natural language processing prowess, has been applied in various fields, including medicine and ophthalmology. However, like any other AI model, it has its limitations, which can impact its effectiveness and applicability in these fields.

One of the significant limitations of the Llama 2 model is its immense computational requirements. The model’s massive neural network architectures, with billions of parameters, demand state-of-the-art hardware and extensive resources for training and fine-tuning. This makes it inaccessible to many individuals and smaller organizations with limited computing power.

## Conclusion

While the Llama 2 model has shown promise in various applications, including medicine and ophthalmology, it is essential to consider its limitations. These include its high computational requirements, lengthy training time, potential for bias, limitations in handling non-English languages, inferior coding abilities, and unclear training dataset. Further research and development are required to address these limitations and enhance the model’s effectiveness and applicability in medicine and ophthalmology.
